# Medical Items and News of Interest to the Physician

**Published:** 1876-04

**Authors:** 


### Medical Items and News,

### For the Use of Physicians.

CULLED FROM THE STANDARD MEDICAL JOURNALS OF THE WORLD.

NOTE.—These formulæ are intended only for the regular practitioner of medicine, and should be employed only by him, or under his immediate supervision.

**Local Anæsthesia,**

May, it is said, be obtained by rubbing for a minute the part upon which it is desired to operate with a mixture of powdered camphor, two and a half drachms, and sulphuric ether, five drachms.

**Thread Worms.**

The oxyuris vermicularis, or seat worm, may readily be dislodged from its favorite habitat in the rectum, by the injection of two to three ounces of ol. morrhuæ, repeated once or twice.—*Philadelphia Med. Times.*

**Offensive Discharges from Cancer, etc.**

To counteract these, washing the parts with a weak solution of sulphurous acid, bathing them with terebene and with a solution of chloral (ʒ i to ℥ iv.), have been recommended.

**To make Court-Plaster Adhere.**

The adhesiveness of court-plaster is much increased by moistening the back of the plaster, after being applied, with a little glycerine; or by adding about 10 per cent of glycerine to the water used for moistening the face of the plaster itself.

**Hiccough.**

Dr. Simon, of Chicago, reports in the *Chicago Jour. and Exam.*, that a patient who had hiccoughed for twenty-six hours, and had been subjected to various treatments without success, was relieved almost instantaneously by inhaling three drops of the amyl nitrite.

**Poisoning by Carbolic Acid.**

A case is reported in the *Medical Times and Gazette*, of a woman in Liverpool, forty years of age, who had swallowed nearly a teacupful of crude carbolic acid. She was seen by Mr. E. S. Wood about twenty minutes after she had taken the dose. He immediately used the stomach pump, and washed out the stomach several times with tepid water. He then injected a pint of olive oil into the stomach, and applied mustard poultices to the legs and precordial region. She was removed to the Northern Hospital, and after about a month's treatment with milk, barley water, lime water and ice, she ultimately recovered.

**Carbolic Acid as an Anthelmintic.**

A Vienna medical journal states that in a case of tænia, this acid was given at first in doses of six grains four times a day; but this proving ineffectual, two grains were ordered every hour, with the result of expelling the worm, head and body, on the third day.

**Post-Partum Hemorrhage.**

Dr. W. Donovan (*London Obstetrical Journal*, August, 1875), says that in cases of flooding after delivery, where ergot has failed, a full dose of tincture of cannabis indica (M. xx.) has in every instance checked the flow in a few moments. It also has the power of controlling and relieving metrorrhagia and profuse menstruation in a marked degree.

**Palpitations of the Heart.**

A correspondent in the *Union Med.*, August 21, calls attention to the fact that palpitations, when not depending upon organic disease, may be almost immediately arrested by bending the head downwards and allowing the arms to hang pendant. The effect is even still more rapidly produced by holding the breath for a few seconds while the body is in this bent position.—*Medical Times, London.*

**Croton-Chloral in Facial Neuralgia.**

Dr. F. B. Lee reports the case of a lady, æt. 32, who had suffered for years from attacks of facial neuralgia, had had several teeth extracted, had been blistered behind the ears, and had tried numerous other means without avail. He prescribed croton-chloral in three grain doses every four hours. After the third dose, perfect ease was experienced, and, although three months had elapsed, there had been no return of the disease.—*The British Medical Journal.*

**Neuralgia.**

In *The Lancet* of November 6, 1875, Spencer Thompson, M. D., writes favorably of the employment of the tincture of Gelsemium sempervirens in neuralgias. The remedy has been used by Dr. Thompson, or under his authority, in at least forty cases, the class most benefited by it being those in which the branches of the trifacial are involved, particularly the inferior maxillary. It is most especially useful when the pain is referred to the teeth or alveoli. Dr. Thompson thinks that the doses generally prescribed are too small, and invariably prescribes twenty minims of the tincture as a first dose, to be repeated in an hour and a half, if relief does not follow. He has rarely had occasion to order a third dose.

**Blistering Substance.**

Dr. Henderson substitutes pure phenic acid for cantharides as a blistering substance; he considers this method the most rapid and least painful, besides not exposing the patient to the cystitis that cantharides causes; it can be applied to the renal region in certain forms of nephritis.—*Union Medicale.*

---

**Cancer.**

In *The British Medical Journal* of November 27th, John H. Wood, M. D., reports a case of cancer of the œsophagus and cardiac orifice of the stomach, in which the symptoms were, for a time, very much relieved by the use of citric acid in large doses, combining it, on Dr. Sidney Ringer's plan, with wine of ipecac in minim doses.

---

**Carious Teeth.**

Dr. Lardier advises, in *L' Union Medicale*, to drop some collodion into carious teeth, after the latter have been cleaned and dried. The liquid collodion exactly fills the cavity of the tooth; the ether evaporates, and narcotizes the nervous twigs. By solidifying, the collodion protects the tooth from the contact of the air. Dr. Lardier states that he has thus succeeded in relieving many patients.

---

**Topical Application in Painful Dentition.**

℞

Syrup of tamarinds, ℥ iiss.

Infusion of saffron, ℈ ii.

Honey, ℥ iiss.

Tinct. (essence) of vanilla, gtt. iv.—M.

Rub gently over the gums with the finger or rag. An application of a similar character is the following:

℞

Saffron, (powdered) 4 to 6 grs.

Honey, 2 to 3 drachms.

Glycerine may be substituted for the honey.

---

**Jaborandi and Atropia.**

Dr. F. V. Greene, *U. S. N.*, says:— "The opportunity of testing the antagonism between atropia and jaborandi being afforded by a patient whose pupil had been dilated with atropia for opthalmoscopic examination, about a grain of a soft extract, made by evaporation of the tincture of jaborandi, was rubbed up with a little glycerine, and painted around the orbit. In half an hour the pupil had contracted very considerably, and at the end of two hours it had returned to its natural size. Since then, I have also tried the effect of the above extract on the pupil in its natural condition, and in four out of five cases found that it produced more or less contraction."

**Chronic Hoarseness.**

In chronic hoarseness arising from thickening of the vocal cords and adjacent membrane, the ammoniated tincture of guaiacum is often a very efficacious remedy. It may be mixed with equal parts of the syrup of senega, and a teaspoonful of the mixture given two or three times daily.—*Am. Practitioner.*

---

**Hydrocele.**

Dr. Lubin, observing the frequent lumbar pain consequent upon the use of the ordinary injections in this affection, was led to use a formula similar to the following:

℞

Tinct. iodinii comp., fl. ℥ iii.
Chloroformi, fl. ʒ iiss.

In a number of cases in which this mixture has been used, no pain whatever followed its injection. —*L' Union Med. du Canada.*

---

**Russian Cure for Drunkenness.**

H. Haurowiz says that for some time past *Herba serpylli* (wild thyme) has been used with great success to effect a permanent cure for drunkenness; in case of a relapse (only after years), a short treatment will effect a cure again. The treatment consists in making an infusion of wild thyme ($1\frac{1}{2}$ oz. to $1\frac{1}{2}$ pints), and give the patient a teacupful every half hour. The next day it is given every two hours, and then four to six times a day until the cure is complete, which generally takes from two to three weeks. The effects are in the following order: vomiting, diarrhœa, increased urine, strong transpiration; then, generally, increased appetite and craving for acidulous beverages. Diet: easily digested food, and lemonade and other acidulous liquids.—*American Journal of Pharmacy.*

---

**Itch.**

℞.

Styrax,
Flor. sulphur.,
Cret. præpar., āā 16 parts.
Green soap,
Axung, āā 32 parts. M.

This ointment is of a greenish yellow color, of good consistence, and of an agreeable odor.

The patient applies the ointment at night before going to bed, taking good care to rub it in thoroughly over those parts where the acari are usually found. These inunctions are repeated for two consecutive days; the patient can go about his business during the day; at the end of three days the patient takes a full bath. For infants at the breast, an equal part of simple cerate is added to the ointment.—*Dr. Weinberg.*

**Fissure of the Anus.**

M. Van Holsbeck states that he has succeeded in curing anal fissures which had resisted the division of the sphincter, with the following application. Dissolve one part of tannic acid in 16 parts of glycerine.

A tent wet with this preparation is to be introduced into the rectum night and morning. The bowels are to be kept open.—*Revue de Therapeutique.*

---

**Malaria.**

Dr. Jos. Berens reports in *The Medical Times* four cases in which good results followed the internal adminstration of chloroform in "chills and fever."

He calls attention to the absolute desuetude into which this remedy has fallen, together with the incontrovertible fact that the potency of quinia is not invariably sustained.

In the cases reported the remedy was given in twenty drop to drachm doses hourly for three to six hours preceding the expected attack.

---

**Ergot--New Preparation of.**

A very active preparation of Ergot, which is particularly adapted for subcutaneous injection, is suggested by Dr. Wernich, of Berlin, who proposes to exhaust the drug with ether, strong alcohol, and finally with water. The infusion is then dialized through parchment paper, and the solution evaporated. This extract, after acidulation with sulphuric acid, was mostly soluble in alcohol, and when again carefully neutralized by soda, yielded all its active properties to weak alcohol.—*Apotheker Zeitung.*

---

**Ergotin in Croupous Pneumonia.**

Dr. Wycisk, acting on the princple that this drug contracts the vessels and so prevents exudation from them, has treated six cases of croupous pneumonia in this way, and gives the following report: In one such case, marked by an excessive highly albuminous expectoration, it ceased entirely two hours after the administration of the drug in powders of nine grains each every quarter of an hour. The abundant rales in both lungs soon diminished, so that there was only a slight crepitation at the original focus of the disease. These good effects lasted for two days. Two relapses occurred, however, but the same good effect was again obtained by the administration of the ergot. After the second relapse, ten drop doses of the tincture of ergot were given four times daily, until convalescence was fully established. In the five other cases the ergot was used early, and none ended fatally, none became chronic, and none left appreciable deposits behind them; in all of them the exudation was decidedly checked by the ergot. It is, however, held to be a dangerous remedy when the lungs are considerably infiltrated, where there is emphysema, where the cerebral arteries are weak, and where the patients are feeble or decrepid.—*Allg. Med. Central Ztg.*

---

**Treatment of Eczema in Children.**

Dr. Calpari in the *Bulletin de Therapeutique*, as quoted in *The Doctor*, extols the effect of lime-water in curing eczema of the head and empetigo of the face in children, especially chronic cases which have resisted other treatment, and states that a marked improvement is noticable after using it for eight days. He recommends to take it in quantities varying up to half a pint, according to the age of the patient, and to dust the part with carbonate of magnesia; but the latter he considers necessary only when the secretion is very irritant.

---

**Scarlatina.**

Dr. W. Scott writes to the editor of the *Medical Press and Circular*:—Without, on the present occasion, entering into the interesting question of whether the minute particles of living matter which constitute disease germs consist of animal or vegetable bioplasm, or the various other difficult points regarding the nature of the poisons of infectious diseases, I am desirous to direct the special attention of practical and thoughtful men to the use of the sulpho-carbolate of sodium, both as a curative and prophylactic agent in the treatment and cure of scarlatina. So far as I am aware, there has not been that general attention given to the subject which its vast importance demands. Mr. Crookes, Dr. A. E. Sansom, Dr. Brackenridge, perhaps others, deserve well for what has been already effected, and, so far as my experience goes, I can fully confirm their testimony; but unless a considerable number of the medical profession attend to the matter, noting cases and briefly publishing the results, but little that is certain can be reliably settled. In scarlet fever, diphtheria and measles, the sodium sulpho-carbolate may be given in doses varying according to age, from five to thirty grains four times daily. In a mixture with simple syrup or other such ingredient it is in no way disagreeable. An extended trial is the all-important point at present, and for this time I need only say that I have seen the use of the above named preparation followed by results far exceeding my most sanguine expectations, both in a preventative and curative point of view.—*Med. & Sur. Rep.*

**Calabar Bean.**

Dr. W. Munroe says, in the *Brit. Med. Journal*: I have used the extract of Calabar bean with advantage as a sudorific, in cases of incipient bronchitis, congestion of the liver, phthisis, with dry hot skin, etc., being always able to bring down the temperature two or three degrees, when sufficient doses were used; such dose being, for an ordinary adult, one-fifth, or at least one-sixth of a grain, as a minimum, which can be safely repeated every four to six hours, till the desired effect is produced, so long as the patient is carefully watched.

---

**Habitual Constipation.**

This formula is recommended by Dr. Marchant as a convenient one to avoid the griping sometimes caused by podophyllin, and to exercise a beneficial effect on the condition of the bowels leading to constipation:

℞
Resin podophyll, gr. ij.
Ext. hyoscyami.,
Sapon. Castil, āā, gr. viij.
Div. in pil. xxiv. M.
Sig. One at night.

---

**Pumpkin Seeds as Anthelmintics.**

Some investigations made by M. Heckel respecting the active parts of pumpkin seeds, are reported in the London *Lancet.*

These seeds have been much used of late for the expulsion of the tapeworm, for which purpose they were employed in the early part of the last century. The mode of their administration has hitherto been to give the seeds in large quantities suspended in water, the outer envelope only having been removed. About two ounces of the seed was the ordinary dose. It is probable that so large a quantity contains much inert matter. Some recent observations apparently indicate that the active principle is contained only in the embryo.

To ascertain whether this is the case was the chief object of M. Heckel's observation. He first admistered, in two cases of tænia, about six ounces of the perisperm, tegumentum and testa, a purgative of castor oil having first been administered. The tapeworm was not expelled in any case. In two other cases the membrane surrounding the embryo was given—about half an ounce,—preceded and followed by a dose of castor oil. In each case the tapeworm was expelled entire. Subsequent experiments yielded the same result. This membrane was then carefully examined, and found to consist of two membranes separable by maceration in water. The outer membrane contained a resin in small quantities, (one in seventeen), which M. Hackel believes to be the active agent. He believes that the castor oil acts not only by its purgative effect, but by dissolving the resin and rendering it active. The second membrane contained more chlorophyll than resin. It must not be forgotten that these seeds contain a fixed oil, to which their qualities have been ascribed, and which may be obtained by cold expression from the seeds in the proportion of half an ounce to a pound. This oil has been used with success, in repeated half-ounce doses, in cese of tænia.

---

**Use of Salicylic Acid in Obstetric Practice.**

Professor Crede gives a short note (*Archiv fur Gynak.*, Bd. vii., Heft 3) on the use of salicylic acid in gynæcological practice. He has employed it for the last six months, on the recommendation of Prof. Kolbe, instead of carbolic acid, as a disinfectant for the hands, as a vaginal injection in puerperal women, and for sprinkling over puerperal ulcers, etc. The strength of the solution is from 1 in 300 to 1 in 900, or as a powder mixed with starch, 1 in 5, or it may be used as salicylic-acid wool. Most favorable results have followed its employment, and Prof. Crede desires strongly to recommend its use in midwifery practice. *Obstet. Journ. of Great Britain.*

---

**Acute Rheumatism.**

Dr. Lee has treated twenty-eight cases of acute rheumatism with a solution of one gramme of propylamin and ten of sugar in one hundred and twenty grammes of peppermint-water, of which a tablespoonful was given every two hours; altogether from three to five grammes were so taken by each patient, whose limbs were bandaged with cotton-wool and card-board. All the twenty-eight cases suffered from multiple joint-affections; in fourteen cases the disease disappeared at the first time, in the other fourteen it was recurrent once or repeatedly. Five cases were complicated with slight, five with severe affections of the heart, one with acute œdema of lungs, and one with diphtheria.—All were restored to perfect health and military duty except one. The average duration of the illness in these cases was 17.7 days per head; none were discharged before full recovery was proved by increased weight of body and gymnastic exercises. The effect of propylamin is summed up as follows: 1. The disease becomes very soon subacute, and remains so to the last. 2. The sedative effect on the nervous system is shown by decreased tension in the circulatory apparatus; pulse and respiration become slower, and high fever decreases within thirty-six hours. 3. With at first profuse, then more gentle perspiration, pain decreases very markedly. 4. The color of the skin acquires a peculiar grayish tint.(?) 5. Sleep quickly returns, and is not interrupted by pain. 6. With a cleaner tongue, appetite returns fast. 7. The quantity of urine is not much increased; it is mostly clear and transparent, only slightly acid, and with little sediment. 8. All patients took the drug without dislike; it was never applied externally.—*Medical Times* from *The Lancet.*

### Chorea Treated by Arsenic.

Dr. Eustace Smith strongly expresses the value of arsenic in chorea. The curative power of the drug is greatly increased by administering it in full doses. Children have a remarkable tolerance for it, especially in such a non-febrile affection where there is no increased irritability of the digestive organs. To a child between the age of five and six and twelve, he gives in chorea as much as ten minims of Fowler's solution after each meal. The effect of treatment is seen almost immediately, and it is rare for any of the physiological actions of the drug to be seen. Even severe cases, under this treatment, rarely last longer than a fortnight.

### Treatment of Wounds.

Dr. A. C. Mackenzie, in the *American Journal of the Medical Sciences*, states that he treats wounds with much success with warm water and a balsamic compound. The warm water not only hastens the exit of the disintegrated material, but conveys to the sufferer that soothing effect which heat alone communicates. The formula used is the following:

Balsam fir.
True Venice turpentine.
Oil of sweet almond, āā ℥ ij.

Add carbolic acid ℥ss., previously dissolved in ℥ ij. warm glycerine. M.

Apply with a flat camel's-hair brush, and inject into the interstices of the wound with glass syringe, having previously cleansed the wound with very warm water and bulb syringe.

Warm water is applied *ad libitum*, and the diseased or injured portion is enveloped in flannel cloths, which have been immersed in water as hot as can be borne comfortably.

### Use of Apocynum Cannabinum in Dropsies.

Dr. Walter Lambert writes to the *Canada Lancet* that during a practice of nineteen years he has often prescibed milk-weed for the relief of dropsies, and with success. He says:—"I always employ the root, and have it dug fresh from the earth, and advise a strong infusion to be made, of which the patient is directed to drink freely, so as to procure two or three watery evacuations from the bowels during the day. It is true that it has sometimes failed to meet my expectations and wishes, but this might be partially accounted for by the root not having been gathered at the proper season of the year; and in some cases its failure has doubtless been due to the origin of the malady. I have found that it is more likely to prove of service in hepatic and cardiac dropsies, and its virtues to be owing to its alterative action on the glandular system, as well as its hydragogue properties, which will not fail to put in an appearance when the drug is administered in sufficient doses."

### Salicylic Acid in Otorrhœa.

Dr. Curtis Smith, in the *Detroit Medical Review*, recommends the following in otorrhœa:—

Acidi salicylici, ℈ j.

Magnesiæ carb., ℈ ij.

Powder very finely and mix.

After cleaning the ear thoroughly with cotton and a fine probe, he introduces a little of this into a small gum tube and blows it into the ear, being careful to have it deposited on the diseased portion. He relates some striking cases cured by this plan, which is simple, and can be easily carried out by any one. It is always necessary to have the diseased surface thoroughly cleaned of all discharge before blowing the powder into the ear.

### Chorea.

Dr. Fiske, (*Pacific Medical Journal*), reports a case cured by anilin. He remarks:

"The anilin at first produced a little nausea, which soon subsided. After the fourth day of treatment, it was continued with camphor, made into pills one grain each. In two weeks the case was cured. Dr. Justin reports several cases in which like results followed. Dr. Peter Jones, of Terre Haute, Indiana, has also used it with like success in several cases, notably one which had continued for fifteen years. Dr. Aaron Wilson, of Nachitoches, has also used it, especially among the negro population, with marked success. It is best to commence with half-grain doses of the medicine, gradually increasing till two grains are given for a dose. Of the sulphate three or four grains, gradually increased to eight or ten grains, may be used. It frequently produces a blueness of the skin, which soon passes away, and need not create any alarm. It has been so prompt and beneficial in its action in the hands of these gentlemen, that its properties should be further investigated, and if found worthy, it should take a higher place in our list of remedies."

**Discoloration by Oxide of Silver.**

In the *Philadelphia Medical Times*, Prof. H. C. Wood refers to the opinion expressed in his work on therapeutics, that the reason no case of discoloration has hitherto been observed from the use of the oxide of silver, is not that it is not capable of producing such pigmentation, but that it has been so seldom exhibited in sufficient quantity. His remark has elicited a letter from Dr. B. A. Clements, of the U. S. Army, who reports a case in which half-grain (3 centigr.) doses, three times daily, for chronic diarrhœa, was exhibited, with an intermission of two weeks in November, from October 23, to December 18, 1874, thus taking, in all, sixty-three grains (4.07 grammes) in forty-two days. At the last mentioned date it was noted that there was a dark, nearly black line near the teeth on the gums of both jaws, especially the lower, and that a bluish discoloration extended to the entire mucous membrane of the gums and lower lip. The general condition was much improved.

---

**Intestinal Obstructions.**

Dr. Robert Battey strongly urges the persistent use of distensile enemata in cases of intestinal obstruction. He protests against the injunction contained in "Flint's Practice," that "the injections are not to be pushed beyond the point at which they are borne without much suffering," and calls in question the statement that "they will very rarely succeed after the invaginated portion of intestine has become swollen by congestion, and the peritoneal surfaces in contact have become adherent." He details successful cases in which twenty and twenty-five pints of fluid were injected until it had passed through the entire length of the alimentary canal and flowed from the mouth, and believes that in *every case* of intestinal obstruction, either feared, suspected, or known to exist, when their duration does not raise a well-grounded apprehension of gangrene, an anodyne having been premised, "a distensile enema ought to be the *first*, and for the most part need be the *only*, power invoked for the cure."—*Atlanta Med. & Surg. Jour.*

---

**Estimation of Uric Acid.**

The usual method of separating uric acid by hydrochloric acid being sometimes inapplicable, and not sufficiently exact, A. P. Fokker was induced to devise a new one, based upon the insolubility of acid urate of ammonia. It is itself not entirely free from error, but in all cases separates more uric acid than the old one. His process is the following: 100 cc. of urine are rendered strongly alkaline by sodium carbonate. After four to six hours the earthy phosphates are filtered off and washed with hot water. Filtrate and wash-water are then mixed with 10 cc. of saturated solution of ammonium chloride, and left without stirring. After from six to twelve hours, the liquid and the precipitate are removed to a small filter, which has been previously washed with one-tenth hydrochloric acid and weighed. When the fluid has run off, the tube of the funnel is fixed into the neck of a bottle by means of a tightly fitting cork. The filter is now filled up to within half a centimetre of its edge with one-tenth hydrochloric acid, and left to stand some hours.—The funnel is then removed from the cork, the fluid allowed to run off, and the sediment washed till it loses its acid reaction, then dried and weighed. According to the author, the previous separation of albumen may be dispensed with. A correction is, however, required, which consists in the addition of sixteen milligrammes (0.016 gm.) to the amount found for every 100 cc. of urine. The author finds that uric acid, when impure, is quite as soluble as when pure. By this method it is, of course, the impure colored uric acid which is estimated.—*J. Chem. Soc.*, from *Pfluger's Archiv f. Phys.*

---

**Gelsemium.**

Berger (*Centralblatt fur die Med. Wissenschaften*, No. 43, 1875), concludes as follows as to the physiological effects of this drug upon cold and warm-blooded animals respectively: When given to animals of the former kind, gelsemium causes cessation of motion and paralysis of the motor centres of the brain, paralysis of the function of respiration, and increase, followed by depression, of the reflex action of the spinal cord. The excitability of the peripheral motor nerve, and also that of the muscles, is diminished, together with the frequency of the heart's action. The administration of the same drug to warm-blooded animals causes paralysis of the motor centres of the brain, which is preceded by a stage of excitement and paralysis of the respiratory centre in the medulla oblongata. Sensibility remains unimpaired, while reflex irritability is at first increased and finally diminished. Its influence upon the heart's action is not very marked, the slight diminution in the frequency of the pulse appearing to be due to the irritation of the origin of the vagus in the medulla by the imperfectly areated blood. Large doses cause a moderate diminution of the blood-pressure; and when death occurs it is always due to paralysis of the respiratory function. In order to form an opinion as to its therapeutic worth, gelsemium was given to many patients suffering with neuralgic affections of peripheral and central origin. In all these cases the results obtained were in the highest degree unsatisfactory, and it must not be forgotten that the instances of fatal poisoning on record stand in marked contrast to the slight narcotic effects of the drug.—*Medical Times.*

**Chloral as a Surgical Dressing.**

In the last number of the *Journal de Therapeutique*, Prof. Marc See, of the St. Eugenie, advances some remarkable evidence in favor of the great value of chloral as a dressing, employed in the proportion of 1 per cent. in water. He first employed it in a case of diphtheritis of the vulva, and the success was so great that he has since used it in numbers of cases during nearly a year, without ever being disappointed. He has applied it in wounds of bad aspect, disinclined to cicatrize, in contused wounds accompanied by much detachment of soft parts, and actual or threatened mortification, or abundant suppuration. He had injected it into the centres of abscesses and sinuses connected with bone, and has also used it in dressing simple wounds, whether accidental or surgical. In all these cases the result was most satisfactory, without any accident whatever calling for the suspension of the chloral occurring. In patients whose wounds on their entrance were complicated with erysipelas or diffused phlegmon, two or three days' use of the chloral has sufficed to arrest the progress of such complications. After cleansing the wound and its vicinity by means of a little charpie (avoiding the use of sponges), dipped in the solution, (and when the wound is deep and irregular, filling it with this, so that it may gain access to the most remote corners), he covers the whole surface with pledgets of carpie thoroughly imbibed with the chloral, and having covered these with oiled silk, envelopes the whole in a thick layer of wadding, and applies a roller somewhat tightly. The chloral is pleasant to the smell, soils neither the fingers nor the bedding, is not too volatile, and causes no pain on application. It has been of great service in several cases of ozæna without necrosis, and it would be difficult to mention all the applications of which it is susceptible. Whenever fetidity has to be destroyed, fermentation, putrefaction, or the production of vibriones, etc., to be arrested, it fulfills the indication with certainty and inoffensiveness. Its moderate price, also, is a matter of importance in hospital administration, as a kilogramme is to be had for twelve or fifteen francs; and as a litre at 1 per cent. contains ten grammes, the price of which is from twelve to fifteen centimes, next to water itself, it is the cheapest article that can be employed.

At a recent meeting of the Societe de Therapeutique (*Bullet. de Therap.*, July 30), M. Crequy strongly recommended chloral as an injection in ozæna, in the proportion of two parts to 250 of water. He places a caoutchouc tube in the vessel containing the solution, and, raising this above the patient's head, allows the fluid to pass into the nose by siphon action. Several members of the Society testified as to the utility of the solution as an application in scrofulous and fetid ulcers, in the eschars produced by decubitus, etc.

**Rheumatism.**

Dr. J. Russell Reynolds, Professor of Medicine in University College, London, urges this treatment. He says, in a recent lecture, I wish to lay before you some further results of that mode of dealing with the disease in question. You will allow me to remind you that the possibility of relieving acute rheumatism by the tincture of perchloride of iron was suggested to my mind by observing the rapid arrest of certain other "spreading" inflammations, such as erysipelas, diphtheroid and hepatic sore throats, by the administration of this drug; and that I stated at the time, and wish now to repeat the statement, that, in my judgment, the cases that I could then bring before you, and those which I can now submit to your consideration, are not sufficiently numerous to establish a therapeutic position; but that they are, so far as I can see, sufficiently significant to warrant a further trial of a mode of treatment which is certainly better than that which Warren said was all that he knew of that was good for rheumatism, viz., six weeks.

In the front of this paper I wish to state, that very many cases that have been under my care, both in hospital and in private practice, but of which I have no sufficient note, have left upon my mind the strong conviction that those which I am able to bring before you, underrate rather than overrate, the value of the mode of treatment that I have suggested. This, I am convinced, is the case, especially with regard to the time of the relief afforded to spontaneous pain.

The treatment has been generally the administration of the tincture of perchloride of iron, in doses varying from fifteen minims to a drachm, every four hours, with or without twenty or thirty minims of glycerine, and spirits of chloroform. No patient has complained of any discomfort of any kind, which could be referred to the medicine.

He continued quoting a number of cases, illustrative of the benefits of this treatment.—*Med. & Surg. Reporter.*

**Epilepsy.**

Dr. J. Warburton Begbie, of Edinburgh, in the Address on Medicine, at the recent meeting of the British Medical Association, (*British Med. Journal*, August 7th, 1875), stated that "his experience of the use of bromide of potassium in epilepsy has been of the most encouraging description. I have repeatedly witnessed cures in the strictest sense result from this employment. Let me briefly refer to one such.

S. A., a bookbinder, aged 50, for twenty years been subject to severe fits, occurring irregularly by night and by day, often attended by biting of the tongue. The usual interval between the fits had been a fortnight, and on no occasion had a longer period than six weeks elapsed. In January, 1870, this patient, whose mental capacity had at that time become considerably enfeebled, so much so as to make it necessary for him to give up his business, began the bromide of potassium, and continued it for eighteen months without any pause. The dose never exceeded twenty grains thrice daily. The result of this treatment was an entire cessation of the epilepsy; there has been no recurrence of the disease. His mental vigor has returned. He long ago resumed his occupation, and has since been busily engaged in it without any interruption.

"I could multiply instances of this kind; and so, I believe, could many practitioners who, in the treatment of epilepsy with bromide of potassium, have been mindful to adhere to the rule upon which Dr. Reynolds insists, that the remedy 'should not be discontinued in the treatment of a case of epilepsy because of its apparent failure; but that the dose should be gradually increased, and the exhibition of the drug most patiently carried on for a period of many months, or even years.'"
—*Monthly Abstract of Med. Science.*

---

**Scarlatina.**

Dr. Bland, in the *Transactions of the Medical Society of the State of Pennsylvania*, says: "In the treatment of scarlatina anginosa, I must again renew my endorsement of the internal administration of the hyposulphites and carbolic acid. The results obtained in this disease with the above remedies are unprecedented, and give every evidence to warrant their continued use. I recently treated seven cases in one family, four of whom had severe throat-ulcerations. The topical application of carbolic acid and glycerine to the inside of the throat, the hyposulphite of soda internally, with good nutritious diet and febrifuge mixture as required, was the treatment employed, and in eight days the patients were all well. A number of my colleagues have tried the foregoing remedies, and give them their unqualified endorsement."

Dr. John Taylor, of Liverpool, writes to the London *Lancet* as follows: "My plan of procedure is to immerse a night-gown, slit up at the front, in hot water (half a pint to a pint), pure or medicated with a drachm or two drachms of tincture of capsicum, or in the infusion of three or four pods, or in mustard-water, the clear supernatant fluid from a table-spoonful of mustard to a pint of water; extending the gown over the feet by means of a towel immersed in the same fluid; both to be well rung out and suddenly applied, and the patient quickly packed in two blankets previously placed on the adjoining sofa or bed; another blanket or two pillows or an eider-down quilt covering all. Modern experience has witnessed the amazing relief procurable from the wet sheet, in its simple form, in pyrexial and glandular disorders, and from the medicated form in the zymotic and spasmodic affections. In stridulous croup, for instance, I have seen the mustard sheet act magically after other means more orthodox had failed. Its power is also potential in diphtheria, simulating croup, and, in strong doses, in inflammatory croup, sometimes averting the impending tracheotomy knife."

---

**Diabetes.**

The treatment of diabetes with opiates, introduced by McGregor, in 1837, has yielded different results in the hand of different physicians. A writer in the *Chicago Medical Journal* strongly asserts that opium, or morpine rather, exerts a direct influence upon the excretion of sugar, diminishing its quantity or making it disappear entirely. But large doses must be given, and to the administration of too small doses does he attribute the ill success of other practitioners.

He bases these assertions on the clinical observations of fourteen cases, in which satisfactory results were obtained by the employment of large doses of morphine. "In most of the cases the disease had lasted a long while, and visibly influenced the nutrition of the patient. None of the patients were put on an absolute animal diet, but always a small amount of bread was allowed. In all cases a decrease in the quantity of sugar in the urine ensued, sooner or later, upon the administration of morphine, and if the dose was gradually increased, the excretion of sugar ceased temporarily. At the same time the excess of urine decreased, the thirst subsided, and the nutrition of the patient improved. With the most patients, it is true, this improvement lasted only as long as morphine was given; in one case, however, no sugar could be found in the urine eighteen months after the treatment had been stopped. The doses of morphine were gradually increased to as much as three grains daily until the urine did not contain any sugar; were then discontinued, to be given again when the sugar re-appeared to the amount of two or three per cent., or moderate doses of morphine were continued, to keep the excretion of sugar below two per cent." The remedy is very well tolerated by such patients, and its narcotic influence does not appear until very late, but it causes an obstinate constipation, which, however, can be counteracted from time to time, by an enema or the use of rhubarb, or aloe.

This writer does not feel prepared to assert that this treatment can cure every case of diabetes, but he thinks it may always lengthen the life of the patient.

---

**A "Certain" Cure for Rheumatism.**

Judging from his article in the *Wiener Medizinische Presse*, Dr. Franz Heller is an enthusiast in the administration of caustic ammonia in rheumatism. For several years he had been a sufferer from severe muscular rheumatism in the right shoulder; he had taken all the common anti-rheumatic remedies with but little alleviation, when he began to reason that in rheumatisim, as in gout, there may be a uric acid diathesis; he thought that liquor ammoniæ, on account of its rapid volatilization, would be the remedy most readily absorbed, and the most prompt in action. In almost the same moment, in which he took one drop, diluted with water, he felt a complete relief from the pain which had lasted for ten hours; he was now able to move freely the arm, which an instant before, he could scarcely bear to have touched. The remedy, he claims, has proved a positive cure in all recent cases of muscular rheumatism which have fallen under his observation; he cites numerous cases in which relief, as instantaneous as his own, was experienced.

He also observed its effects in several cases of acute articular rheumatism, in two of which six drops sufficed to subdue the pain and swelling within a period of twenty-four hours.

In one case of chronic rheumatism of a finger joint, which had lasted for over half a year, the simple administration of the ammonia completely dispelled the inflammation and pain in the joint within two days.

He then discusses the mode of action of his remedy. "If we consider an excessive acidity as the cause of the rheumatism, we can scarcely claim in the cases in which one drop will instantaneously relieve the pain in recent rheumatism, that one drop was sufficient to counteract the effects of the excess of uric or (according to Fuller), lactic acid.

"Nothing remains therefore but for us to seek for the source of rheumatism in a morbid nervous activity induced by disturbances of nutrition, and to believe that the ammonia acts as a nervine directly upon the nerves."

---

A writer in the *Medical Record*, while making a report of the common practice in the treatment of diseases in a Philadelphia Hospital, gives the following notes:

**Night Sweats of Phthisis.**

Sulphate of atropia is regarded as an excellent remedy for their arrest. It is usually administered in a simple dose at bed-time of $\frac{1}{60}$ of a grain.

Aromatic sulphuric acid.

Niemeyer's pill. Niemeyer's pill and atropia used in conjunction. Oxide of zinc and hyoscyamus were said to be efficient.

The pill called Niemeyer's, or the one he ordinarily prescribed, properly known by the name of Heim's pills, is in general prescription. The formula for the pill is as follows:

℞
Pulv. herb. digitalis, ℈ ss.
Pulv. rad. ipecac.
Pulv. opii puri, āā gr. v.
Ext. heleni, q. s. u. f. pl. No. xx.
Comp. pulv. rad. irid. flor.

S. A pill three times daily. The addition of a scruple of quinine to the above prescription constitutes Niemeyer's modification, and is added for its antipyretic effect. This pill of Niemeyer's is one very commonly prescribed in this hospital in cases of phthisis, and is regarded as a very valuable combination of remedies.

**Hæmoptysis.**

Hypodermic injections of ergotine were mentioned with special favor. The solution used is of such strength that ♏ xv. contain one grain of the ergotine.

Inhalations of the oil of turpentine are frequently employed.

As good success has attended the use of the above remedies as any which have been employed.

**Quinine and Morphine.**

It has been noticed in several cases that when one-quarter of a grain of morphine would not produce sleep, if ten grains quinine were administer- a short time previous to administering the morphine, the morphine would almost invariably act efficiently. This fact was noticed in connection with puerperal cases.

